# Intravenous Sedation in a Patient With Sotos Syndrome and Intellectual Disability: A Case Report

**DOI:** 10.7759/cureus.76555

**Published:** 2024-12-29

**Authors:** Izumi Kuroda, Arisa Fujita, Yoko Okumura, Naoko Tachi, Aiji Sato-Boku

**Affiliations:** 1 Department of Anesthesiology, Aichi Gakuin University, Nagoya, JPN; 2 Department of Anesthesiology, Nagoya University Graduate School of Medicine, Nagoya, JPN

**Keywords:** dental treatment, intravenous sedation, mental retardation, microtia, sotos syndrome

## Abstract

Sotos syndrome is a genetic disorder characterized by distinct facial features, intellectual disability, and overgrowth. In this case, a patient with Sotos syndrome presented with severe intellectual disability, for which general anesthesia was initially considered. However, at the request of the patient’s guardian, the treatment was performed under intravenous sedation. Due to the patient’s severe intellectual disability and microtia, administering intravenous sedation was anticipated to be challenging. Nevertheless, the dental treatment was successfully and safely completed by employing multiple strategies. These included using risperidone as premedication, applying a local anesthetic cream, securing the peripheral venous route after nitrous oxide inhalation, and monitoring respiratory status with a capnometer to adjust sedation levels. This report aims to demonstrate the successful management of intravenous sedation in a patient with Sotos syndrome, focusing on the strategies employed to address the unique challenges posed by severe intellectual disability, behavioral difficulties, and anatomical considerations. By highlighting these approaches, we aim to contribute to the limited literature on sedation management for patients with complex medical and behavioral needs.

## Introduction

Sotos syndrome is an autosomal dominant genetic disorder characterized by facial features, intellectual disability, and overgrowth resulting in height and macrocephaly. The syndrome has an incidence of approximately one in 14,000, and it is not uncommon to encounter Sotos syndrome in daily clinical practice [1-3]. Although anecdotal reports of Sotos syndrome have been published in the literature regarding its clinical manifestations, genetic analysis, and management under general anesthesia, there are no reports of management under intravenous sedation that we are aware of [4,5]. Problems of anesthesia management include difficulty in inducing anesthesia and treatment refusal due to severe mental retardation and upper airway obstruction due to microtia. Microtia itself does not directly cause upper airway obstruction; however, it is frequently associated with craniofacial abnormalities such as mandibular hypoplasia. These structural issues can lead to tongue displacement or airway narrowing, particularly under deep sedation or anesthesia. This relationship warrants careful monitoring during procedures requiring sedation. We have experienced a case in which a patient with Sotos syndrome was managed under intravenous sedation without complications by applying several innovations. This report aims to demonstrate the safe and effective management of intravenous sedation in a patient with Sotos syndrome, focusing on overcoming challenges such as intellectual disability, behavioral difficulties, and anatomical risks. By detailing the strategies employed, this report seeks to contribute to the limited literature on sedation protocols for patients with complex medical and behavioral needs. Written consent was obtained from the patient’s guardian to report this case.

## Case presentation

**Figure 1:**
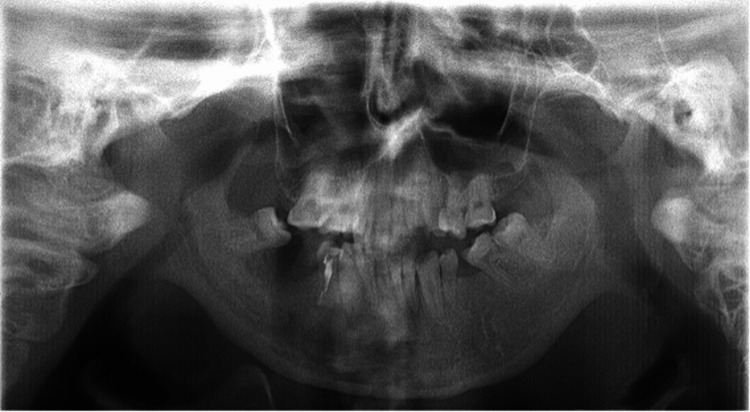
Panoramic radiograph. The patient was uncooperative, making it difficult to take the X-ray, so the image is from seven years ago

The patient was a 27-year-old man who attended Aichi Gakuin University Dental Hospital for treatment. He was 178 cm tall and weighed 50 kg. Conservative dental treatment was planned to address carious teeth in regions 43-78 and L7, as shown in Figure 1.

He had been receiving outpatient dental treatment under nitrous oxide inhalation sedation for five years. Although he underwent regular check-ups only for intraoral examination for three years, carious teeth were discovered that required treatment. His adaptability to dental treatment gradually decreased over the past two years, making treatment difficult even under nitrous oxide inhalation sedation. The patient exhibited severe intellectual disability, with an inability to understand instructions or communicate effectively. Behavioral challenges included treatment refusal, aggression, and dependency on caregivers. These factors necessitated the use of pharmacological and non-pharmacological strategies to facilitate the procedure.

As a result, dental treatment was planned using pharmacological behavior modification methods such as general anesthesia or intravenous sedation. The patient had a history of Sotos syndrome characterized by intellectual disability and auditory hypersensitivity. Because of his intellectual disability, he had difficulty communicating, and his mood swings were such that on some days, he could be treated in the outpatient clinic, while on other days, he refused to go to the clinic altogether. As his refusal increased, he displayed aggressive and harmful behaviors, such as hitting others around him, including his parents. At the age of 25 years, he was hospitalized for two weeks due to increased aggressive behavior toward his parents. Around this time, he lost interest in activities he previously enjoyed and became anxious when his mother was not around, often following her closely. His regular medications included lemborexant 5 mg, bifidobacteria, and risperidone tablet 0.5 mg, with risperidone 0.5 mg taken on an as-needed basis during agitation. He usually lived in an institution, with his mother and an aide from the institution as his primary caregivers. The patient exhibited a peculiar facial appearance associated with Sotos syndrome, along with a small mandible. He underwent atrial septal defect surgery at the age of one year and scoliosis surgery at the age of 18 years. At the age of 19 years, he underwent dental surgery under general anesthesia at Aichi Gakuin University Dental Hospital, during which no perioperative complications were observed.

Initially, a similar dental treatment under general anesthesia was planned for the patient at the age of 27. However, intravenous sedation was ultimately chosen for the procedure during the same year, based on the guardian’s request and the specific circumstances of the treatment. Due to the patient’s behavioral challenges and severe intellectual disability, the preoperative examination was limited to blood testing. The blood test results were within normal ranges, and no abnormalities were identified that would contraindicate sedation or the planned procedure.

**Figure 2:**
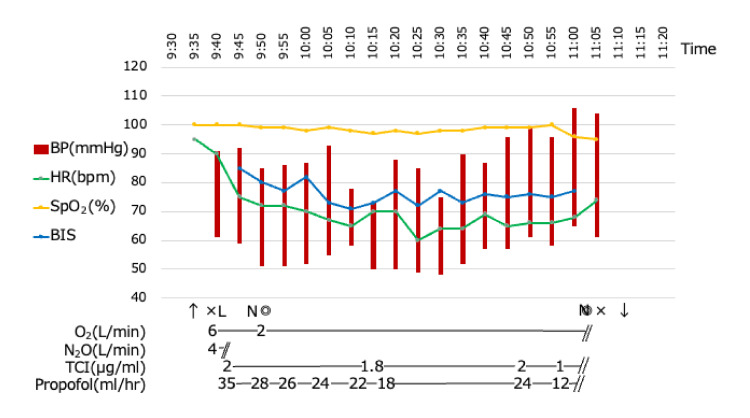
Anesthesia record of reoperation BP: arterial blood pressure; HR: heart rate; SpO_2_: peripheral capillary oxygen saturation; BIS: bispectral index; ↑: entered operating room; ×: start or completion of anesthesia time; L: peripheral IV access secured; N: nasal cannula initiated; ◎: start or completion of the operation; TCI: target-controlled infusion

On the day of the procedure, the patient was administered a premedication dose of risperidone 0.5 mg, which was given one hour prior to his arrival at the clinic. This premedication was intended to manage agitation and facilitate the subsequent steps of the procedure. Next, a lidocaine/propitocaine patch (Emla® patch (Aspen Pharmacare Australia Pty Ltd, St Leonards, NSW)) was applied to his right forearm 45 minutes before he entered the clinic to relieve puncture pain during the securing of a peripheral venous channel. At that time, the patient was very wary of the anesthesiologist, whom he had never met before, and showed aggressive behavior, such as grabbing the anesthesiologist. Immediately after applying the Emla® patch to the forearm, the patient’s forearm was covered with a jacket to divert his attention. The patient could no longer wait in the waiting room for 10 minutes before entering the clinic and was admitted at that time. His mother and an aide accompanied him as he entered the treatment room. The dental chair was placed in a horizontal position, and the patient was asked to lie down. Nitrous oxide inhalation was then administered under the guidance of the attending dentist. After nitrous oxide sedation, a peripheral venous line was established using a 22-G needle at the site of the Emla® patch. Propofol was then continuously administered at a target-controlled infusion (TCI) rate of 2 μg/mL. Sedation levels were adjusted using the bispectral index (BIS). The treatment began once the sedation level reached a BIS of 70. The anesthesia record is presented in Figure 2.

**Figure 3:**
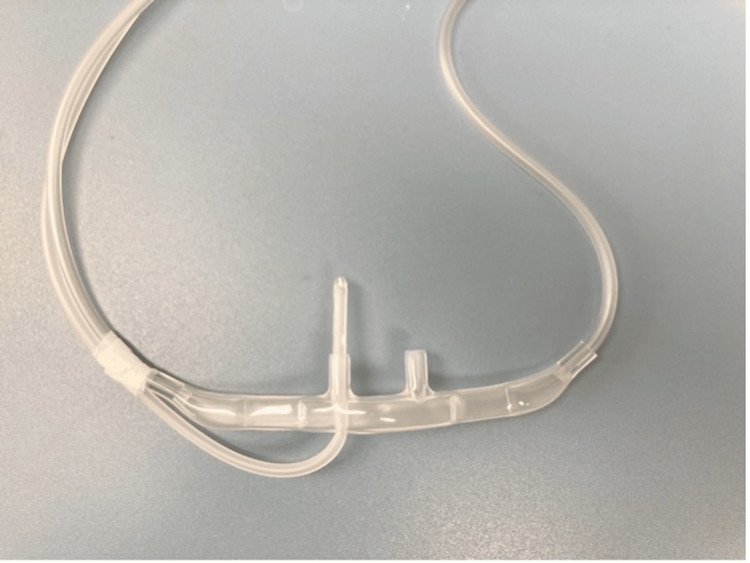
Nasal cannula

Because the patient had a small mandible, a deep sedation level was anticipated to easily cause upper airway obstruction. Therefore, the sedation level was maintained in the BIS 70 range throughout the treatment. The respiratory status was monitored using expiratory gas analysis. A nasal cannula connected to a capnograph via a Top Nelaton® catheter (8 Fr) was used to sample exhaled carbon dioxide (Figure 3). 

During sedation, no symptoms of upper airway obstruction, such as snoring, were observed, and both respiratory and circulatory states remained stable. The anesthesia and procedure durations were 93 minutes and 74 minutes, respectively. The patient went home after awakening with a postoperative recovery score of 10.

## Discussion

Sotos syndrome is an autosomal dominant genetic disorder caused by abnormalities in the nuclear receptor-binding Su(var)3-9, enhancer of zest, and trithorax domain-containing protein 1 (NSD1) genes [1]. It is characterized by a peculiar facial appearance with a large head and prominent forehead, intellectual disability, and macrocephaly due to overgrowth. These features are present in >90% of patients with Sotos syndrome [1]. Accelerated bone aging, heart and kidney abnormalities, seizures, and scoliosis are observed in 15%-30% of patients and are highly variable in type and severity [1]. Although some reports on the clinical manifestations and genetic analysis of Sotos syndrome, as well as on the management of general anesthesia, have been conducted, no reports have investigated intravenous sedation to our knowledge [4,5]. In this patient, refusal of treatment due to difficulty in the induction and maintenance of anesthesia due to severe intellectual disability and upper airway obstruction during sedation due to a small mandible were considered when performing intravenous sedation. More than 90% of patients with Sotos syndrome have intellectual disabilities. The severity varies from mild to severe, but the mean intelligence quotient (IQ) is estimated to be about 61 (range 37-101 in a cohort of 52 patients) [6]. The patient had a severe intellectual disability and had difficulty communicating. The patient also strongly refused treatment, making the outpatient treatment difficult, and had aggressive behavior toward others, including his parents. The first issue was whether the patient could enter the anesthesia room on the day of anesthesia management. The patient takes risperidone tablets 0.5 mg, a serotonin and dopamine antagonist, during agitation. Risperidone blocks dopamine 2 (D2) receptors in the brain and suppresses positive symptoms caused by hyperactivity of the dopamine nervous system, blocks serotonin 2 (5-HT2) receptors, and improves negative symptoms. Considering the possibility that the patient might have difficulty entering the examination room due to strong refusal, risperidone tablet 0.5 mg was administered as a preanesthetic medication. Since risperidone reaches its maximum blood concentration in about one hour postadministration, risperidone was administered one hour before entering the clinic. The mother, the primary caregiver for the patient, and her aide also entered the room with the patient. As a result, the patient’s admission to the clinic went smoothly.

Next, since the patient could inhale nitrous oxide without resistance in the outpatient clinic, the patient was administered nitrous oxide inhalation, and after sedation, we secured a peripheral venous route and switched to intravenous sedation. In the outpatient clinic, the dental chair was placed in the horizontal position, and the same was done in the clinic where the sedation was performed. The principal dentist for disability dentistry with whom we had established a relationship was asked to guide the patient to the dental chair and place a mask over his face to induce nitrous oxide inhalation. The anesthesiologist adjusted the nitrous oxide inhalation concentration, and the principal dentist held the mask and encouraged the patient to take deep breaths. As a result, the patient was able to lie down on the dental chair by himself, and the nitrous oxide inhalation was administered smoothly. The nitrous oxide concentration was 40%.

After confirming that nitrous oxide was effective and the patient was sedated, a peripheral venous line was secured. The pain of peripheral venous catheter insertion is associated with pain and distress for many patients, and pain relief should be practiced in both adults and children [7]. The Emla® patch was preapplied to the planned puncture site to prevent body movement due to puncture pain. A significant analgesic effect of the Emla® patch at the time of puncture compared to the placebo and a local cooling device has been observed [8,9]. In the present study, the application of the Emla® patch in combination with the effect of nitrous oxide allowed the insertion of a peripheral venous catheter without any body movement, with only light manual restraint during the puncture.

In general, patients who are intellectually challenged are unable to understand the treatment and its benefits, resulting in inappropriate behavior toward dental treatment, for which general anesthesia or sedation is chosen as a method of pharmacological behavior modification. In particular, patients who are intellectually challenged often require deep sedation to control their unconsciousness. Patients with intellectual disabilities have been reported to have a lower BIS during general anesthesia than normal patients, and the amount of propofol required to achieve the target BIS was lower [10-12]. Therefore, intravenous sedation of patients who are intellectually challenged will tend to increase the depth of anesthesia even when a normal dose of sedative is administered. Intravenous sedation is a particularly effective method of anesthesia in patients who are mentally challenged. Thus, intravenous sedation should be used with caution, especially in patients at risk for upper airway obstruction, and Sotos syndrome is a condition that should be treated with caution during intravenous sedation because it is often associated with microtia mandibularis.

In this case, the patient also had a small mandible, and the depth of anesthesia was thought to cause a slight upper airway obstruction during treatment due to tongue root subsidence. However, no signs of upper airway obstruction, such as snoring, were observed during the procedure. This can be attributed to meticulous sedation management, including continuous respiratory monitoring using capnometry and maintaining a controlled sedation depth with a BIS level of around 70.

In this case, the patient was receiving oral risperidone as a preanesthetic and inhaled nitrous oxide as an induction agent and propofol as a maintenance agent; thus, the interaction of these agents should be considered. In addition, evaluating the degree of sedation in patients with intellectual disability is more difficult than usual because they tend to deepen the depth of anesthesia and have difficulty understanding the patient’s condition through communication. Therefore, we decided to evaluate the level of sedation objectively using a BIS monitor. In addition, the TCI system was used for continuous propofol administration under the guidance of the BIS monitor. The administration of propofol by TCI has been reported to reduce the dose of propofol during sedation, thereby preventing overdose [13]. Propofol also allows for easier adjustment of sedation levels compared to benzodiazepine sedatives such as midazolam.

Again, in this case, particular attention should have been paid to the upper airway obstruction associated with the small mandible. To prevent upper airway obstruction, respiratory depression can be prevented and detected early by intermittent observation of thoracic movements, monitoring of breath sounds as needed, and the use of expiratory end carbon dioxide partial pressure monitoring (capnometry). In particular, ventilation monitoring by expiratory gas analysis is recommended to enhance the safety of intravenous sedation. This made it possible to adjust the sedation level so that respiratory depression did not occur on the monitored capnometer waveform. As described above, using TCI while checking the BIS value, the depth of sedation was accurately evaluated, and the waveform of the capnometer enabled the early detection of respiratory depression and the determination of an appropriate dose of propofol. As a result, no upper airway obstruction was observed during treatment, and the BIS level was maintained in the 70s with propofol TCI at 2 μg/mL during treatment. The BIS level of 70 corresponds to a state of moderate sedation, similar to a score of 3-4 on the Ramsay sedation scale. In addition, the patient was successfully treated without moving or waking up during the treatment.

Patients with intellectual disability have difficulty understanding the dental treatment, and maladaptive behavior during treatment may make the treatment difficult. In addition, the burden on patients and their families increases when multiple treatments are required depending on the nature of the treatment; thus, pharmacological behavioral adjustment is often chosen as a management method. General anesthesia allows immobilization without the patient’s understanding and cooperation and ensures safe and reliable treatment. It is also less likely to cause trauma to the patient. However, patients are subjected to high stress, both physically and mentally, due to the unusual hospital environment and procedures necessary for anesthesia management, such as induction of anesthesia and extubation, as well as restraints required for these procedures. The patient’s family also bears a heavy financial burden in terms of accompanying the patient to the hospital and paying for hospitalization and general anesthesia. Conversely, intravenous sedation is less expensive than general anesthesia, and the patient can go home on a day trip without hospitalization. Because of the patient’s severe intellectual disability, we initially considered general anesthesia for the management, but after considering the wishes of the patient’s guardian, the patient’s characteristics, and the nature of the procedure, we decided to manage the patient with intravenous sedation. In this case, the patient’s doctor asked the patient about what the patient was familiar with and what he could tolerate, and the anesthesia management was aimed at minimizing the patient’s stress in cooperation with personnel and medical staff with whom a rapport had been established, which led to safe management without problems.

## Conclusions

Intravenous sedation was performed on a patient with Sotos syndrome, a patient who is severely intellectually challenged with difficulty communicating. However, considering the patient’s characteristics, anesthesia was safely administered without complications by carefully obtaining information from the physician in charge of the patient, collaborating with the key person and the physician in charge of the patient, and using various techniques for anesthesia management. In conclusion, while this case highlights effective sedation management strategies for a patient with Sotos syndrome, the generalizability of these findings is limited by the unique characteristics of the patient and the single-case design of this report.
